# Low correlation between biometric parameters, cardiovascular risk factors and aortic dimensions by computed tomography coronary angiography

**DOI:** 10.1097/MD.0000000000021891

**Published:** 2020-08-28

**Authors:** Ernesto Forte, Bruna Punzo, Marco Salvatore, Erica Maffei, Stefano Nistri, Carlo Cavaliere, Filippo Cademartiri

**Affiliations:** a IRCCS SDN, Naples; b Department of Radiology, Area Vasta 1/ASUR Marche, Urbino; c Cardiology Service-CMSR Veneto Medica, Altavilla Vicentina, VI, Italy.

**Keywords:** anthropometrical features, aortic diameter, aortic root, ascending aorta, computed tomography coronary angiography, sinotubular junction

## Abstract

To analyze the relationship between aortic measures and biometric parameters in a large cohort of consecutive patients undergoing computed tomography coronary angiography.

1170 patients (717 men/453 women) performing computed tomography coronary angiography for coronary evaluation were retrospectively evaluated. Aortic diameters and areas were measured at reproducible anatomic landmarks, perpendicular to the axis of vessel, at the level of the aortic root (AoR), the sinotubular junction (STJ), and the tubular ascending aorta (TAo). Biometric parameters and cardiovascular risk factors were recorded.

The average values of AoR, STJ, and TAo were 35.63 ± 5.00 mm, 30.56 ± 4.82 mm, 35.07 ± 5.84 mm. Hypertension was significantly associated with aortic dimensions.

Aortic measures were significantly different between men and women (37.56 ± 4.77 mm vs 32.58 ± 3.68 mm for AoR, 31.88 ± 4.84 mm vs 28.47 ± 3.98 mm for STJ and 35.93 ± 5.86 mm vs 33.70 ± 5.54 mm for TAo) (*P* < .001) and linearly increased with age. Low Spearman correlation coefficients were found and the correlation of TAo diameters with age displayed the highest values (*ρ* = 0.372 for male and *ρ* = 0.373 for female, *P* < .001). Multiple linear regression analysis models were compared by *R*^2^. The best model used body surface area (BSA) and age as independent variables and TAo diameter as dependent variable (*R*^2^ = 0.29 for AoR; *R*^2^ = 0.21 for STJ, and *R*^2^ = 0.20 for TAo).

In conclusion, in our population low correlation between aortic dimensions and biometric parameters highlights the difficulty of identifying normal ranges, as well as issues related to normalization using conventional biometric parameters.

## 1 Introduction

Accurate and reproducible measurements of aortic diameters are essential for the diagnosis, classification, and follow-up of aortic pathologies and to decide how/when to perform follow-up, prevention strategies, and select candidates to surgery.^[1]^ In the past decade, there have been remarkable advances in non-invasive imaging of aortic disease. In this field, many imaging techniques have been employed for the assessment of aorta and its segments such as transthoracic echocardiography (TTE), transesophageal echocardiography, magnetic resonance imaging(MRI), computed tomography (CT), and conventional angiography. The most frequently used non-invasive technique in clinical practice is CT, as it is readily available and enables, thanks to the recent development of multidetector technology, the simultaneous evaluation of the aorta and coronaries.^[2–4]^

Values for normal ranges have been first established by ultrasound^[5–12]^ while more recent studies used CT.^[13–16]^

To date it has been very difficult to identify a single method able to provide the concept and the ranges of variability of normal ascending aorta. In previous studies^[5–16]^ the correlation between aortic sizes and biometric parameters has been exploited by echocardiography or non-contrast electocardiogram (ECG) triggered CT^[13]^ in selected population (healthy subjects with no risk factors or with non-obstructive coronary artery disease).

In this study, we aimed to analyze in a large cohort of consecutive patients undergoing computed tomography coronary angiography (CTCA) the relationship between aortic dimensions and demographics, biometric parameters and risk factors, using different approaches.

## 2 Material and methods

One thousand one hundred seventy (1170) consecutive patients, respecting the inclusion criteria mentioned below (717 men/453 women; mean age ± standard deviation = 62.70 ± 12.80 years) and referred to CTCA for suspected coronary artery disease, were retrospectively evaluated. All demographics risk factors and patient relevant clinical data were prospectively gathered from medical records, such as age, gender, weight, height, and cardiovascular risk factors including family history of aortic disease, smoking status, diabetes, dyslipidemia, hypertension, and obesity. Dyslipidemia, diabetes, and hypertension were defined according to the current guidelines.^[17–19]^ All patients underwent CTCA for coronary artery assessment which includes in the dataset ascending aorta. All patients gave informed consent for the investigation and the study was approved by the Institutional Ethics Committee (patients were enrolled in a prospective local registry of CTCA).

Inclusion criteria are stable heart rate with sinus rhythm and ability to hold breath for at least 12 seconds. Exclusion criteria are known aortic disease, bicuspid aortic valve, previous coronary revascularization, previous acute myocardial infarction or severe heart failure, severe renal impairment (serum creatinine >2 mg/dL), atrial fibrillation, thyroid disorders, unstable clinical condition, known allergy to iodinated contrast agents and pregnancy.

A dose of 5 mg atenolol was administered intravenously before the scan if the patients’ heart rate was >65 beats per minute. In addition, all patients received 0.8 mg of isosorbide dinitrate sublingually immediately before the scan.

### 2.1 Scan protocol

All scans were performed on a 64-slice multidetector CT scanner (Sensation 64 Cardiac; Siemens, Germany). The angiographic study was preceded by an unenhanced acquisition to evaluate the distribution and amount of coronary calcium. The calcium score scan was performed using prospective ECG-gating with the following parameters: collimation 20 × 1.2 mm, gantry rotation time 330 ms, feed/rotation 4.8 mm, effective slice width 3 mm, increment 1.5 mm, kV 120, effective mAs 150.

The CTCA was performed after intravenous administration of 80 to 100 mL of high iodine concentration contrast agent (Iomeprol 400 mg I/mL-Iomeron-400, Bracco, Milan, Italy) at a rate of 4 to 5 mL/s followed by a 40 to 50 mL saline chaser at the same rate. A bolus-tracking technique was used to synchronize the arrival of contrast in the coronary arteries and the initiation of the scan. The following parameters were set: retrospective ECG-gating with prospective modulation of the dose, collimation 32 × 2 × 0.6 mm, gantry rotation time 330 ms, feed/rotation 3.84 mm (pitch 0.2), effective slice width 0.75 mm, reconstruction interval 0.4 mm, medium-smooth B30f reconstruction kernel, kV 120, mAs 700 to 900 (depending on patients’ features).

The temporal windows for ECG-gated retrospective reconstructions were set at the end-diastolic (−300 ms, −350 ms, and −450 ms before the next R wave) and end-systolic (+225 ms, +275 ms, and +325 ms after the previous R wave) phases.

### 2.2 Image evaluation

**Figure 1:**
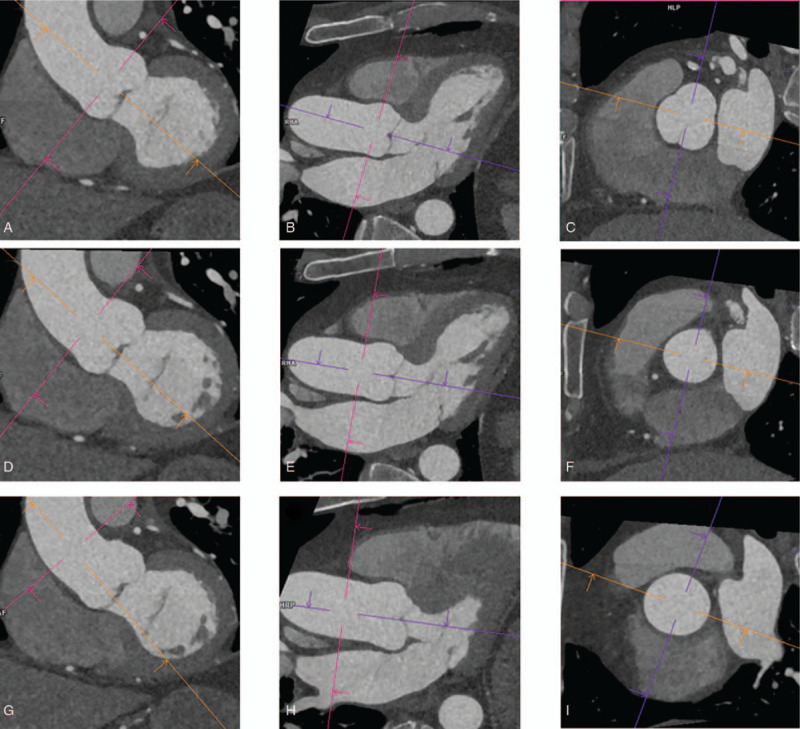
(A, D, G) Left ventricle output tract and (B, E, H) 3 chamber view multiplanar reformation (MPR) showing the level of aortic root (AoR), sinotubular junction (STJ), and tubular ascending aorta (TAo). (C, F, I) Cross-sectional images.

Image data were analyzed in consensus by 2 experienced operators on a dedicate offline workstation (MMWP; Siemens). Aortic diameters (technique used: inner-to-inner) and areas (technique used: intimal lumen contour) were measured on diastolic dataset at conventional and reproducible anatomic landmarks, perpendicular to the axis of vessel. The aortic root (AoR) was measured as cusp-to-commissure in correspondence of the maximum diameter, the sinotubular junction (STJ) was measured at the narrowest level in the transition of AoR to the ascending aorta and the tubular ascending aorta (TAo) was taken at the level of the right pulmonary artery. Measurements are represented in Figure 1. Thereafter, numerical values were used for statistical analysis.

### 2.3 Statistical analysis

Statistical analysis was performed using R Core Team (version 3.03 Austria, Vienna). Continuous variables were expressed as mean ± standard deviation or standard error. Data were tested for normality through the Shapiro–Wilk test. For comparison between 2 groups, the unpaired *t*-test or the Mann-Whitney test was chosen according to variables’ distribution. The 1-way analysis of variance or the Kruskal–Wallis test were used for comparison among 3 groups for parametric and non-parametric variables, respectively. In case of statistical significance, the Bonferroni post-hoc test was chosen. Inter-observer reproducibility was evaluated by intraclass correlation coefficient. Categorical variables were expressed as percentage and compared using the Chi-square test or the Fisher exact test. The Spearman correlation coefficient was used to analyze the relationship between aortic measurements and anthropometric features. Multiple linear regression analysis was performed in order to compare our data with published results.^[1,6,7]^ Aortic sizes were used as dependent variables and biometric parameters such as age, body surface area (BSA), and height as independent variables. In each analysis, *R*^2^ was determined to give the proportion of the variability in the aortic size attributable to demographic variables. A *P*-value < .05 was considered for statistical significance.

## 3 Results

Demographic and clinical characteristics of the study population, stratified by gender, are reported in Table 1. The average values of AoR, STJ, and TAo were 35.63 ± 5.00 mm, 30.56 ± 4.82 mm, 35.07 ± 5.84 mm.

**Table 1:**
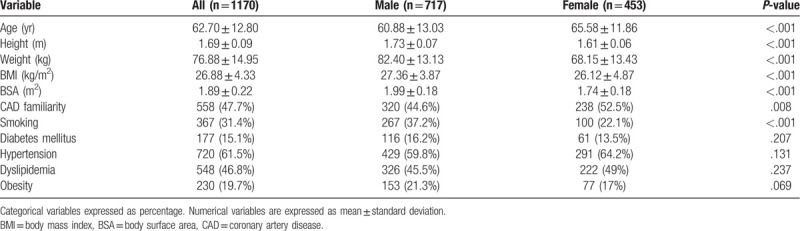
Baseline population characteristcs.

Among cardiovascular risk factors, familiarity, dyslipidemia, hypertension, and obesity were significantly associated with aortic dimensions (familiarity: *P* = .001 for AoR and *P* < .001 for STJ and TAo; dyslipidemia: *P* = .024 for AoR, *P* = .019 for STJ and *P* = .033 for TAo; hypertension: *P* = .002 for AoR and *P* < .001 for STJ and TAo; obesity: *P* = .030 for AoR, *P* = .002 for STJ and *P* = .001 for TAo). Smoking status and diabetes were not significantly associated with aortic dimensions (smoking status: *P* = .091 for AoR, *P* = .361 for STJ, and *P* = .516 for TAo; diabetes: *P* = .745 for AoR, *P* = .690 for STJ, and *P* = .255 for TAo).

**Figure 2:**
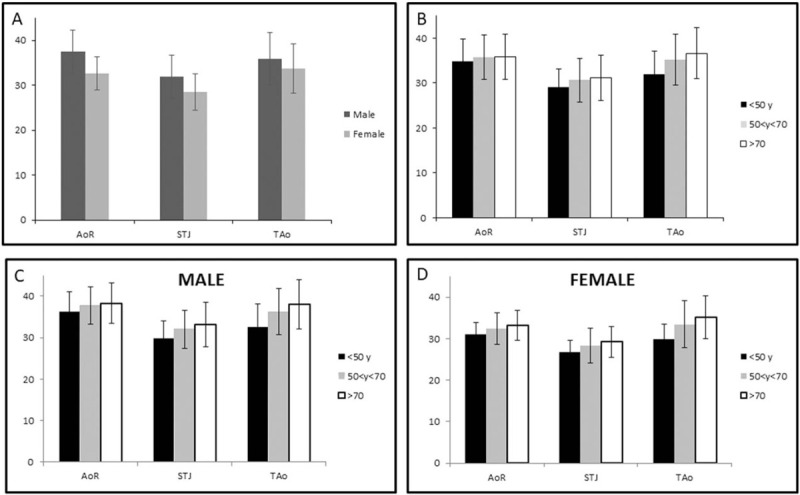
(A) Aortic diameters in Male (n = 717) and Female cohort (n = 453). (B) Patient population stratification based upon age (<50 yr old, 50 < yr < 70 and >70 yr old). (C and D) Intra-cohort comparison of aortic diameters, considering sex and age-based stratification. AoR = aortic root, STJ = sinotubular junction, TAo = tubular ascending aorta.

Height, weight, body mass index, and BSA were significantly larger in men than in women (*P* < .001). Intraclass correlation coefficient was 0.971 *P* < .001 for AoR; 0.970 *P* < .001 for STJ and 0.976 *P* < .001 for TAo. Aortic measures were significantly larger in men as compared to women (37.56 ± 4.77 mm vs 32.58 ± 3.68 mm for AoR; 31.88 ± 4.84 mm vs 28.47 ± 3.98 mm for STJ and 35.93 ± 5.86 mm vs 33.70 ± 5.54 mm for TAo) (*P* < .001) (Fig. 2A) and increased linearly with age (Fig. 2B, C, and D). Our population was stratified in 3 groups according to age: group A (n = 205) included subjects younger than 50 years old (<50 y), group B (n = 617) those 50 < y < 70 and group C (n = 348) subjects >70 y. Statistical analysis revealed that AoR and STJ diameters in men were significantly lower when comparing group A versus group B (*P* < .001) and group A versus group C (*P* < .001) but no statistically significance was found comparing group B versus group C (*P* = .625 for AoR and *P* = .112 for STJ). Instead, women presented statistical significance when comparing AoR and STJ in group B versus group C (*P* = .001 for AoR and *P* *<* .001 for STJ) and group A versus group C (*P* = .024 for AoR and *P* = .003 for STJ) but not group A versus group B (*P* = .141 for AoR and *P* = .057 for STJ). As for TAo diameters, a statistically significant difference was found between all groups (*P* < .001).

Indexing diameters by BSA eliminated the significant difference between men and women at the level of the AoR (*P* = .49), but not in the other segments (*P* = .01 for STJ and *P* *<* .001 for TAo) (Table 2). On the other hand, indexing by height and body mass index resulted in gender-independent diameter of the TAo (*P* = .48 and *P* = .50, respectively).

**Table 2:**
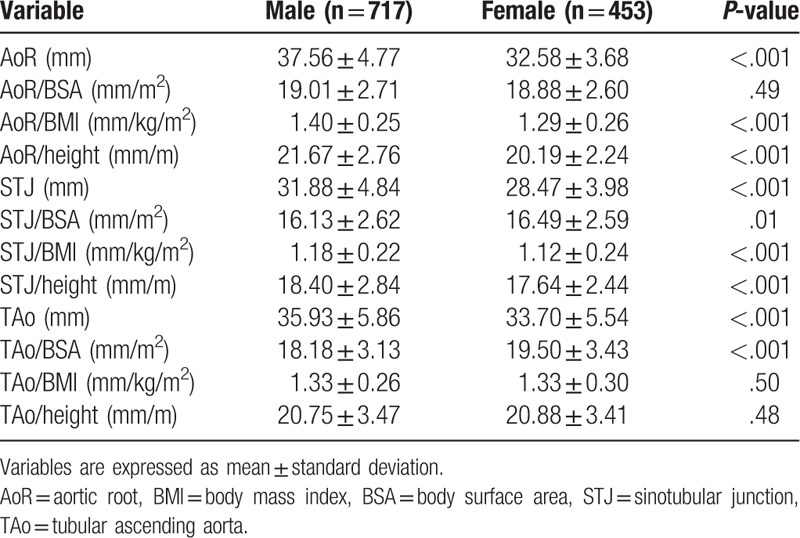
Gender based comparison of indexed aortic diameters.

There were weak correlations between the aortic diameters/areas and biometric parameters in both genders (Table 3). TAo correlation coefficients with age displayed the highest values (*ρ* = 0.372 for men and *ρ* = 0.373 for women, *P* < .001).

**Table 3:**
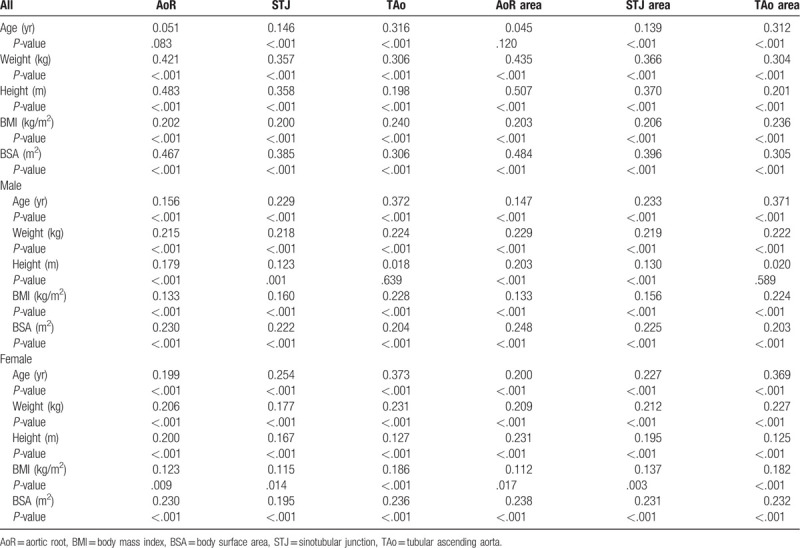
Spearman correlation coefficients.

Multiple linear regression analysis was performed using BSA and age as independent variables (Table 4) and height and age as independent variables (Table 5). Aortic diameters were independently associated with age, gender, and BSA. This model performed better in terms of *R*^2^ values (*R*^2^ = 0.29 for AoR; *R*^2^ = 0.21 for STJ; and *R*^2^ = 0.20 for TAo) as compared to the model including age, gender, and height (*R*^2^ = 0.28 for AoR; *R*^2^ = 0.19 for STJ; and *R*^2^ = 0.16 for TAo).

**Table 4:**
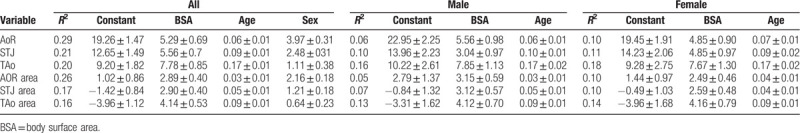
Multiple linear regression analysis with age and BSA.

**Table 5:**
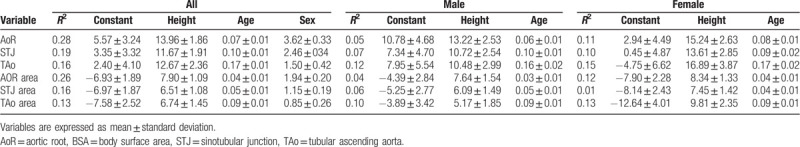
Multiple linear regression analysis with age and height.

We conducted a sub-group analysis on patients without any risk factor (n = 101; *F* = 34; *M* = 67) and the model including age, gender and BSA confirmed the previous results (*R*^2^ = 0.25 for AoR; *R*^2^ = 0.26 for STJ; and *R*^2^ = 0.42 for TAo) as compared to the model including age, gender, and height (*R*^2^ = 0.25 for AoR; *R*^2^ = 0.25 for STJ; and *R*^2^ = 0.38 for TAo).

## 4 Discussion

The assessment of aortic dimensions is of particular importance especially for the diagnosis and prognosis of vascular pathologies such as aortic aneurysms, bicuspid aortic valve, and genetic syndromes. An accurate evaluation of the aortic size is crucial in patients carrying aortic dilation in which a surgical treatment may be planned. Therefore, disease progression needs to be monitored over time since increasing aortic diameters increase the likelihood of aortic dissection, rupture or, eventually, death.

### 4.1 Imaging techniques

Various imaging modalities are used for aortic evaluation such as TTE, transesophageal echocardiography, MR, and CT.^[20]^

Many literature reports published reference values of aortic annulus, AoR, STJ, and TAo established by ultrasound.^[6–12,21–24]^ Nevertheless, some concerns arise about the accuracy and reproducibility of measurements since TTE is an operator dependent modality with limited 3-dimensional capabilities and potential issues with acoustic window that could lead to over/under-estimation. MR and CT provide a more accurate evaluation of aortic size eventually highlighting findings masked on TTE. MR supplies a broad range of sequences (black blood or bright blood), contrast media injection can be introduced in the scan protocol to obtain 3D angiograms, and, thanks to its high contrast resolution, it well delineates the blood flow and the vessel wall.^[25]^ CT is the preferred modality for the diagnosis, risk stratification, and management of aortic disease. Despite the use of ionizing radiations and the need for contrast media, it depicts with high spatial and geometric accuracy the aorta and its branches, well identifying both the lumen and the vessel wall as well as the presence of calcifications, atheroma, endoleaks, and dissection flaps.^[26,27]^

It is necessary to adopt standardized measurements to better assess changes in aortic size over time and avoid erroneous findings of arterial growth.^[5]^ All imaging modalities entail inherent limitations and the use of ECG-gating during acquisition is essential to properly evaluate the aortic walls and avoid misdiagnosis.^[20]^ Due to intrinsic differences between methods,^[10]^ it is recommended that identical imaging technique should be used for serial measurements and the measure be taken on 3D data when available and using always the same technique.^[28–30]^

### 4.2 Cardiovascular risk factors

In healthy adults, aortic diameters do not usually exceed 40 mm and taper gradually downstream.^[5]^ Due to variations in size with patient age, gender, and body surface area, having a single diameter cutoff for abnormal diameter is frequently inaccurate. However, the traditionally accepted values for the upper limits of normal diameter for the sinuses of Valsava and the STJ are 4 cm and 3.6 cm for males and 3.6 cm and 3.2 cm for females respectively.^[31]^ Indeed, it is commonly acknowledged that several cardiovascular risk factors interfere with aortic size such as gender, age, cholesterol, or blood pressure.^[6–12,23,24,32,33]^ Among these, hypertension influences the enlargement of more distal aortic segments^[18]^ while aortitis and genetic aortopathies may affect the proximal ascending aorta. The progression of aortic dilation with age is thought to be related to a higher collagen to elastin ratio, along with increased stiffness and pulse pressure.^[8,11,32–34]^ For this reason, several authors reported aortic reference values evaluated in selected patients stratified, for example, by age and sex.^[1,6,7,10,13–16,20–22,35]^ Nevertheless, the analyzed cohorts often comprise healthy subjects without coronary artery disease or cardiovascular risk factors.

According to these studies, in our population women were older, shorter, and weight less than men. As discussed by Nevsky et al,^[16]^ this result likely reflects the increased risk of disease for men for a given age.^[36]^ The analysis in groups stratified according to age clearly indicate that aortic dimensions in men predominantly increase faster until 50 years and afterward more gradually while in women there is a turnaround with a slow increase until 50 years becoming noticeable later. These results can be interpreted, at least partly, in light of the protective effect of estrogens before menopause in women. In agreement with literature data,^[8,23,24]^ all aortic diameters were significantly larger in men than women.

### 4.3 Biometric parameters

In line with Muraru and colleagues,^[1]^ indexed values comparison denote a greater influence of BSA on AoR diameter while TAo is mainly influenced by height.

As compared to the aforementioned studies,^[1,13,16]^ the correlations between aortic dimensions and biometric parameters in our population provided low values both in male and female.

### 4.4 Linear regression models

Multiple linear regression analyses reflect this key point. In fact, aortic diameters were independently associated with age, gender, and BSA with a decreasing trend in *R*^2^ ranging from AoR to TAo. When the effect of age and risk factors was removed, an increased trend in *R*^*2*^ was observed both in male and female. In detail, the sub-analysis conducted in patients without risk factors, even if it constitutes a smaller sample, revealed that *R*^2^ values slightly increase. The highest correlations and *R*^2^ obtained for TAo underline that age and anthropometrical features have more influence on ascending aorta than AoR and STJ, reflecting a physiologic and mechanic cause. Moreover, in light of our results normal reference values and indexed parameters should be carefully considered when examining patients.

### 4.5 Limitations

Our study has some limitations: though patients were enrolled consecutively it is not a prospective study; we evaluated aortic measures only in 1 cardiac phase, in end-diastole, so the aortic distension during cardiac cycle was not examined. Moreover, we only focused on aortic size not analyzing its association with coronary artery disease.

### 4.6 Future directions

There is currently the need to switch to investigational and observational studies and registries which should be able to use many more parameters and most probably other parameters. With this we mean that there is a need to introduce more complex assessment of available parameters and more parameters that are going to become what is currently defined as radiomics.

## 5 Conclusions

In conclusion, in our population CT aortic dimensions measured at AoR, STJ, and TAo showed low correlations with biometric parameters when we considered the potential influence of patients’ cardiovascular risk factors. This finding in a large cohort of consecutive patients highlights the difficulty of identifying normal ranges, as well as issues related to normalization using conventional biometric parameters.

## Author contributions

1. Guarantor of integrity of the entire study: Filippo Cademartiri.

2. Study concept and design: Ernesto Forte, Carlo Cavaliere, Filippo Cademartiri.

3. Literature research: Bruna Punzo.

4. Clinical study: Erica Maffei, Filippo Cademartiri, Stefano Nistri.

5. Experimental studies/data analysis: Erica Maffei, Filippo Cademartiri, Stefano Nistri.

6. Statistical analysis: Ernesto Forte

7. Manuscript preparation: Ernesto Forte, Bruna Punzo, Carlo Cavaliere.

8. Manuscript editing: Marco Salvatore, Erica Maffei, Stefano Nistri.
